# Effect of a maternal and newborn health system quality improvement project on the use of facilities for childbirth: a cluster‐randomised study in rural Tanzania

**DOI:** 10.1111/tmi.13220

**Published:** 2019-03-11

**Authors:** Elysia Larson, Anna D. Gage, Godfrey M. Mbaruku, Redempta Mbatia, Sebastien Haneuse, Margaret E. Kruk

**Affiliations:** ^1^ Department of Global Health and Population Harvard T.H. Chan School of Public Health Boston MA USA; ^2^ Department of Biostatistics Harvard T.H. Chan School of Public Health Boston MA USA; ^3^ Ifakara Health Institute Dar es Salaam Tanzania; ^4^ Tanzania Health Promotion Support Dar es Salaam Tanzania

**Keywords:** maternal and newborn health, quality, utilisation, Tanzania, cluster‐randomised controlled trial, evaluation, santé maternelle et néonatale, qualité, utilisation, Tanzanie, essai contrôlé randomisé en grappes, évaluation

## Abstract

**Objectives:**

Reduction in maternal and newborn mortality requires that women deliver in high quality health facilities. However, many facilities provide sub‐optimal quality of care, which may be a reason for less than universal facility utilisation. We assessed the impact of a quality improvement project on facility utilisation for childbirth.

**Methods:**

In this cluster‐randomised experiment in four rural districts in Tanzania, 12 primary care clinics and their catchment areas received a quality improvement intervention consisting of in‐service training, mentoring and supportive supervision, infrastructure support, and peer outreach, while 12 facilities and their catchment areas functioned as controls. We conducted a census of all deliveries within the catchment area and used difference‐in‐differences analysis to determine the intervention's effect on facility utilisation for childbirth. We conducted a secondary analysis of utilisation among women whose prior delivery was at home. We further investigated mechanisms for increased facility utilisation.

**Results:**

The intervention led to an increase in facility births of 6.7 percentage points from a baseline of 72% (95% Confidence Interval: 0.6, 12.8). The intervention increased facility delivery among women with past home deliveries by 18.3 percentage points (95% CI: 10.1, 26.6). Antenatal quality increased in intervention facilities with providers performing an additional 0.5 actions across the full population and 0.8 actions for the home delivery subgroup.

**Conclusions:**

We attribute the increased use of facilities to better antenatal quality. This increased utilisation would lead to lower maternal mortality only in the presence of improvement in care quality.

## Abstract (fr)

**Objectifs:**

La réduction de la mortalité maternelle et néonatale exige que les femmes accouchent dans des établissements de santé de haute qualité. Cependant, de nombreux établissements offrent une qualité de soins sous‐optimale, ce qui peut expliquer l'utilisation moins généralisée des établissements. Nous avons évalué l'impact d'un projet d'amélioration de la qualité sur l'utilisation des établissements pour l'accouchement.

**Méthodes:**

Dans cet essai randomisé en grappes mené dans quatre districts ruraux de Tanzanie, 12 cliniques de soins primaires et leurs zones de recrutement ont bénéficié d'une intervention d'amélioration de la qualité consistant en une formation au cours du service, une supervision par un encadrement et un accompagnement, un appui en infrastructure et des relations avec les pairs tandis que 12 établissements et leur zone de recrutement ont servi de contrôles. Nous avons procédé à un recensement de tous les accouchements dans la zone de recrutement et utilisé une analyse de la différence des différences pour déterminer l'effet de l'intervention sur l'utilisation des établissements pour l'accouchement. Nous avons effectué une analyse secondaire de l'utilisation chez les femmes dont l'accouchement précédent avait eu lieu à domicile. Nous avons également investigué les mécanismes permettant d'accroître l'utilisation des établissements.

**Résultats:**

L'intervention a entraîné une augmentation du nombre de naissances dans les établissements de 6,7 points de pourcentage par rapport à une de référence base de 72% (intervalle de confiance à 95%: 0.6–12.8). L'intervention a augmenté de 18.3 points de pourcentage l'accouchement dans un établissement pour les femmes ayant accouché à domicile précédemment (IC 95%: 10.1–26.6). La qualité prénatale a augmenté dans les établissements d'intervention, les prestataires effectuant 0.5 action supplémentaire sur l'ensemble de la population et 0.8 action pour le sous‐groupe des accouchements à domicile.

**Conclusions:**

Nous attribuons l'utilisation accrue des établissements à une meilleure qualité prénatale. Cette utilisation accrue ne ferait baisser la mortalité maternelle que si la qualité des soins s'améliorait.

## Introduction

After two decades of global policy and action focused on increasing the proportion of births occurring in health facilities, many populations have seen a shift in delivery location to health facilities 1, 2. However, gaps in facility utilisation for childbirth persist in many regions, particularly throughout sub‐Saharan Africa (SSA) 1. High quality facility‐based care, with good access to emergency obstetric care, has the potential to reduce preventable maternal and newborn mortality and morbidity 3, 4. Improving utilisation, therefore, remains an important, unfinished global priority.

Maternal characteristics, such as education, socio‐economic status, and parity, are often cited as reasons for incomplete facility utilisation 5. Similarly, many efforts to increase facility utilisation for childbirth have focused on the demand side; for example, providing travel vouchers, fee exemption, community education and text message reminders 6, 7, 8. While some demand‐side interventions have been successful in increasing facility utilisation, recent evidence suggests that quality plays an important role in motivating (when quality is strong) or dissuading (when quality is weak) utilisation 9, 10.

The quality of care available to women in many low‐ and middle‐income countries is both low and inequitably distributed 11, 12, 13, 14. Studies in SSA have demonstrated women's preference for high‐quality obstetric care, including a desire to deliver with competent and kind healthcare providers 15, 16, 17. In addition, their judgment of quality of care is related to both how they are treated and the comprehensiveness of services 18. Women frequently bypass lower‐level clinics in favour of health centres and hospitals, where they are likely to receive better quality care 11, 19, 20, 21. Interventions focused on improving the quality of maternal and newborn healthcare are on the rise, with success measured by changes in quality or health outcomes 22, 23, 24. However, because of women's preference for, and ability to distinguish high quality care, facility utilisation may also increase when quality improves. To date there is little evidence from randomised studies of the effect that quality may have on utilisation.

In this study we assess the impact of a quality improvement program on facility utilisation for delivery, using a cluster‐randomised controlled study in Tanzania. Given that the implementation evaluation did not find meaningful improvement in quality of obstetric care, we explored alternative pathways between program investments and utilisation of services. Broadly, these findings are relevant for understanding how investments in the health system may influence user behaviour.

## Methods

### Study setting, design and participants

The maternal and newborn health quality improvement (MNH+) study was implemented in four rural districts of Pwani Region, Tanzania: Bagamoyo, Kibaha Rural, Kisarawe, and Mkuranga. The study is registered through ISRCTN (http://www.isrctn.com/ISRCTN17107760). Government‐managed primary care facilities (i.e., dispensaries) and their official catchment areas define the clusters for this study. Facilities were eligible for inclusion in the MNH+ study if they were supported for prevention of maternal to child transmission of HIV care by a local non‐governmental organisation (Tanzania Health Promotion Support, THPS) and had at least one skilled healthcare provider at the start of the start of the study (e.g. a nurse or clinical officer). From the eligible facilities, within each of the four districts the six facilities with the highest volume of deliveries between January and June 2011 where chosen, resulting in 24 study facilities. At baseline, three of six facilities within each district were randomly selected to receive the MNH+ intervention (12 facilities total). Random selection was conducted by drawing facility names from a hat in the presence of representatives from the research team and the regional medical office.

The intervention included three components to improve facility quality: infrastructure improvement (facility upgrades and ensuring basic equipment and supplies), provider training and supervision (continuing medical education, supportive supervision and mentoring), and peer outreach to promote facility utilisation for childbirth within the official catchment communities. Implementation of the intervention began in June 2012; by July 2013 the full intervention was underway and continued until after endline data collection was completed.

Women were eligible to participate if they had delivered a child 6 weeks to 1 year prior to interview, lived in the catchment area of a study facility, and were at least 15 years of age. All survey participants provided written, informed consent, or assent together with permission from a guardian in the case of minors, prior to participation. Ethics review boards in both Tanzania, Ifakara Health Institute and the National Institute for Medical Research, and in the U.S., Columbia University and Harvard University, approved this study.

### Data collection and variables

The baseline round of data collection was conducted from 13 February to 28 April 2012. Midline data were collected from 3 February 2014 to 31 March 2014. The endline round was conducted from 20 January to 7 April 2016. At baseline and endline the study team enumerated all households in each catchment area and invited all eligible women to participate in the study. During the midline data collection, the study team again enumerated all households in each catchment area to create a list of eligible women. From this list, a simple random sample of 60% of women stratified at the facility level was invited to participate in the study.

Trained Tanzanian research assistants conducted the household survey in Swahili using hand‐held tablets. Tanzanian research staff translated the survey to Swahili and back‐translated to English by consensus. In our prospective analysis plan we determined a limited set of predictors of utilisation that would be included in statistical analyses. To select these predictors we started from Anderson's utilisation model of predisposing characteristics and a review of recent literature 20, 25, 26. The individual‐level demographic factors included women's religion, age, marital status, parity, educational achievement, primary occupation, and season of delivery. We constructed an indicator of relative household wealth using principal components analysis of an 18‐question asset index 27. To indicate a woman's exposure to mass media we constructed an additive media index utilising three questions that are also measured in the DHS: frequency of exposure to radio, newspapers, and TV (range 0–9). Finally, as an indicator of potentially changing community‐level physical access to the health system, we included an indicator for whether a woman's village had a paved road.

To assess potential pathways through which the intervention could affect utilisation (analyses described below) we collected information on antenatal care (ANC) quality, perceived obstetric quality, link between facility and community, and payment for obstetric care. For ANC quality we asked women if they had received the following during antenatal care and created an index of nine items: weight measured, height measured, blood pressure measured, urine sample collected, blood sample collected, tetanus injection administered, iron supplements provided, antimalarial medications provided and counselled on pregnancy complications. We also asked them to rate their perceived quality of ANC and separately their perceived quality of obstetric care on five‐level Likert scales ranging from poor to excellent. We categorised high‐perceived quality as a rating of ‘very good’ or ‘excellent’. To assess the link between the facility and community we asked women if they had heard of a quality improvement program in their local facility. Finally, to measure their payment for care we asked women how much they paid for all services, including any informal payments, or tips.

### Statistical analysis

Completed surveys were formatted as CSV files and imported into Stata version 14.1 for cleaning and analysis. The primary study outcome was facility utilisation for childbirth, which was measured as the proportion of women in the facilities’ official catchment area whose most recent delivery occurred in a healthcare facility. Women who reported delivering on the way to a facility were removed from the analysis (72 women, 1.2%). We conducted descriptive statistics of the primary outcome as well as the level of facility women delivered at (i.e. dispensary, health centre, or hospital), women's place of delivery for her delivery prior to the index child (the *index child* is defined as the woman's most recent delivery), and demographic and household characteristics.

To illustrate the changing patterns of utilisation we plotted the proportion of deliveries that occurred at any health facility with lowests trends for each month. The graph includes all months where the entire month fell within eligibility for participation in the study (April 2011–January 2012, April 2013–January 2014, April 2015–January 2016).

To measure the effect of the MNH+ intervention on facility utilisation for childbirth we conducted a difference‐in‐differences analysis. This analysis controls for both differences between utilisation patterns between facilities at baseline and changing patterns over time that are external to the intervention, but consistent across the region. In all models a fixed effect for district was included to account for stratification of the study facilities by district during the design phase. We used generalised estimating equations with an exchangeable correlation structure and a log link to estimate risk ratios for all models 28. The robust sandwich estimator was used to account for clustering at the facility level.

In order for the difference‐in‐differences coefficient to be a valid estimator of causal effect, the parallel trends assumption must be satisfied; that is the assumption that in the absence of the intervention the intervention and control facilities would have had similar increases in facility utilisation for childbirth. We tested this assumption by creating a dataset of repeat cross‐sectional data for each month where we had birth data for at least 10 women prior to start of the intervention. We conducted several additional robustness checks and sensitivity analyses, which are described in the Appendix S5. These included different evaluation models (e.g. a post‐only analysis), and different statistical methods (e.g. Fischer Permutation tests).

Recognising that women who have previously delivered at home have an increased likelihood of subsequent home delivery 29, we conducted a secondary analysis in which our sample was restricted to women whose most recent delivery prior to her *index child* was a home birth to test whether the intervention had a differential effect on this group (Appendix S3). This difference‐in‐differences analysis of women with previous home births was not pre‐specified but added when baseline data indicated high overall utilisation relative to hypothesised levels. This allowed us to explore effects of the intervention on a group that has historically been difficult to reach with safe delivery coverage expansion efforts. We found evidence of modification of the effect of the intervention by place of a woman's previous birth and therefore conducted a stratified analysis by women whose delivery prior to her index child was a home birth versus women whose prior delivery was a facility birth or were primiparous.

**Figure 1:**
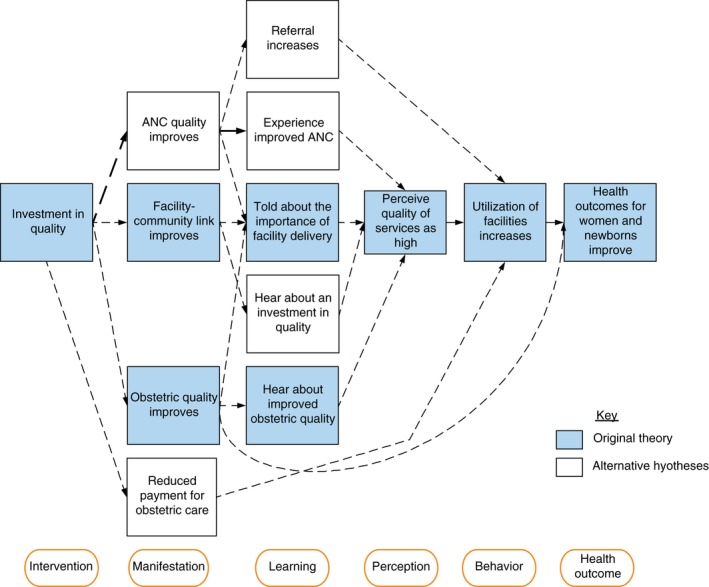
Conceptual framework for pathways through which investment in quality could affect facility utilisation for childbirth. [Colour figure can be viewed at http://wileyonlinelibrary.com]

We explored four pathways through which an investment in quality could lead to increased utilisation of facilities for childbirth: ANC quality, perceived obstetric quality, link between facility and community and payment for obstetric care (Figure 1). Our model is informed by prior theoretical work as well as empirical evidence demonstrating women's stated and revealed preferences for high quality care 5, 17, 25, 30, 31. Because the implementation evaluation found that the intervention did not affect the quality of delivery care, we do not present these results here. For each pathway we explore first the effect of the intervention on the intermediary outcome, then the association between the intermediary outcome and facility utilisation for childbirth at baseline.

## Results

In 2012 the study team enumerated 30 076 households, identifying 3238 eligible women and interviewing 3019 (response rate of 93.2%). The flow diagram can be found in Appendix S1. In 2016 the study team enumerated 36 390 households, identifying 3779 eligible women. We interviewed 3575 women, resulting in a response rate of 94.6%. Of those women, 3146 had delivered their child in the 6 weeks to 1 year prior to delivery and were eligible for inclusion in this analysis. The resulting sample size was 6083 observations and 5992 unique women. 91 women delivered and were interviewed twice in the study period.

The average age of respondents was 27 years old in the intervention and control groups at baseline and endline. The proportion of women who had completed any secondary school was seven percentage points higher at endline than baseline in both the control (17%) and intervention (15%) groups. At endline 91% of women reported living in a household with a mobile phone and nearly a quarter reported having electricity (Table 1). Covariates appear largely balanced at baseline.

**Table 1:** Descriptive statistics of women delivering in Pwani region, Tanzania (2011–2012 and 2015–2016)

	Baseline		Endline	
	Control (*n* = 1586)	Treatment (*n* = 1393)	Control (*n* = 1739)	Treatment (*n* = 1365)
Demographics				
Age (mean)	27.2	27.0	27.2	27.0
Education (categorical)				
No formal	25%	28%	20%	21%
Some primary	13%	13%	11%	9%
Completed primary	51%	51%	51%	54%
Any secondary	10%	8%	17%	15%
Farmer or homemaker	82%	82%	78%	77%
Muslim	79%	84%	71%	78%
Married or living with partner	82%	83%	82%	81%
Household assets				
Media index (mean)^a^	3.37	3.18	3.38	3.25
Household wealth: richest 20%^b^	22%	18%	31%	25%
Mobile phone	73%	74%	91%	91%
Electricity	7%	5%	23%	25%
Consumes > 2 meals per day	90%	90%	90%	88%
Delivery characteristics				
Primipara	25%	23%	27%	28%
Birth during harvest season	17%	17%	20%	22%
Previous delivery at facility	59%	62%	76%	76%
Delivery at facility^c^	72%	72%	81%	85%
Community characteristics				
Village has paved road	29%	43%	35%	43%
District				
Bagamoyo	42%	42%	53%	53%
Kibaha rural	11%	10%	12%	8%
Kisarawe	25%	25%	20%	25%
Mkuranga	22%	22%	15%	13%

a Media index range (0, 12).

b Wealth index constructed using baseline asset weights for both baseline and endline cohorts.

c Dependent variable.

**Figure 2:**
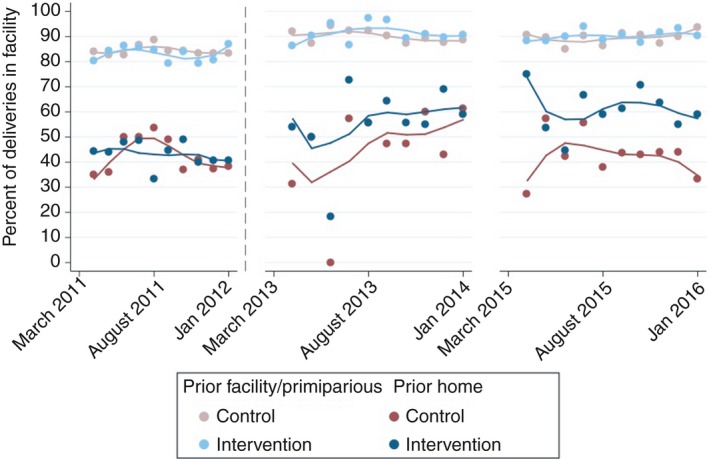
Trends in facility deliveries; proportion of deliveries occurring at any health facility by month stratified by previous home delivery. Notes: Previous home delivery group are women whose birth prior to the index child was at home. Previous facility/primiparious group are women who either were primiparious for the index child or delivered their last child in the facility. Solid lines represent lowess trends. The intervention began June 2012 and is denoted by a vertical dashed line. [Colour figure can be viewed at http://wileyonlinelibrary.com]

At baseline, 72.3% of women living in control catchment areas delivered their most recent child in a health facility, compared to 71.7% of women living in intervention catchment areas (the intracluster correlation was 0.10). The proportion of women delivering in health facilities increased at endline to 81.1% of women in control catchment areas and 85.3% of women in intervention catchment areas. The intervention therefore led to a relative increase in facility deliveries of 10% (RR: 1.10; 95% confidence interval (CI): 1.00, 1.21). The adjusted relative risk is 1.08 with a 95% CI of 0.98, 1.19 (Table 2). This translates to an absolute increase of 6.7 percentage points (95% CI: 0.6, 12.8) in a linear model and 6.1 percentage points (95% CI: −0.8, 13.1) in an adjusted linear model. Our interrupted time series analysis suggested that the parallel trends assumption was satisfied: the relative difference in the trend of facility birth between the intervention and control group prior to the intervention was 0.99, *P*‐value: 0.488 (Figure 2 and Appendix S5).

**Table 2:** Effect of MNH+ intervention on facility utilisation for childbirth, unadjusted and adjusted difference‐in‐difference analyses

	Full population						Previous home birth			Previous facility birth or primiparous		
	Unadjusted			Adjusted			Unadjusted			Unadjusted		
	RR	95% CI	*P*‐value	aRR	95% CI	*P*‐value	RR	95% CI	*P*‐value	RR	95% CI	*P*‐value
Difference‐in‐difference												
Effect of MNH+	1.10	1.00, 1.21	0.041	1.08	0.98, 1.19	0.112	1.42	1.17, 1.71	<0.001	1.05	0.98, 1.12	0.165
Year indicator	1.13	1.06, 1.21	<0.001	1.12	1.05, 1.20	<0.001	1.10	0.96, 1.26	0.187	1.07	1.01, 1.12	0.013
Intervention indicator	0.90	0.77, 1.05	0.177	0.91	0.81, 1.03	0.132	0.86	0.66, 1.12	0.265	0.98	0.88, 1.08	0.660
Participant characteristics												
Age (years)				1.00	1.00, 1.00	0.695						
Religion (Muslim)				1.01	0.98, 1.03	0.614						
Education												
No formal				Reference								
Some primary				1.05	0.99, 1.11	0.115						
Completed primary				1.11	1.07, 1.15	<0.001						
Any secondary				1.11	1.06, 1.16	<0.001						
Birth during harvest season				1.03	0.99, 1.08	0.094						
Primipara				1.08	1.04, 1.12	<0.001						
Media use index				1.01	1.00, 1.02	0.002						
Wealth quintile												
Lowest				Reference								
Lower middle				1.08	1.04, 1.13	<0.001						
Middle				1.21	1.12, 1.30	<0.001						
Higher middle				1.23	1.11, 1.35	<0.001						
Highest				1.31	1.17, 1.46	<0.001						
Farmer or homemaker				0.95	0.93, 0.98	<0.001						
Married				1.00	0.97, 1.03	0.994						
Community characteristics												
Village has a paved road				1.12	0.96, 1.31	0.145						
District												
Bagamoyo	Reference			Reference			Reference			Reference		
Kibaha Rural	1.29	1.13, 1.47	<0.001	1.18	1.02, 1.36	0.027	1.27	0.87, 1.85	0.209	1.09	0.96, 1.24	0.201
Kisarawe	1.11	0.98, 1.26	0.115	1.07	0.91, 1.26	0.408	1.32	0.92, 1.88	0.130	1.06	0.94, 1.20	0.330
Mkuranga	1.30	1.14, 1.49	<0.001	1.31	1.13, 1.52	<0.001	1.54	1.05, 2.25	0.027	1.18	1.05, 1.33	0.005
Observations	6083			6003			1375					

The predictor of interest is the ‘effect of MNH+’, which is the interaction between time (year dummy variable) and intervention indicator (whether or not the facility was in the intervention group). We used generalised estimating equations with an exchangeable correlation structure and a log link to estimate risk ratios. This accounts for clustering at the facility level. District is included in both the unadjusted and adjusted models to account for the study design: facilities were stratified by district prior to randomisation. Previous home birth refers to the birth immediately prior to the index child in women who reported two or more births.

We assessed the pattern of deliveries by facility level over time and found that the decline in home deliveries was explained by an increase in deliveries at both the intervention facility and higher‐level facilities, such as health centres and hospitals (Appendix S6). When looking specifically at utilisation of the study facility as the outcome of interest (compared to other facility or home delivery), the relative risk was 1.04 (95% CI of 0.76, 1.43).

Of the women interviewed at baseline who had previously delivered a child, 39.4% reported delivering their previous child at home (40.5% in control facilities and 38.1% in intervention facilities). These women were older, had less education and lower wealth than the full sample (Appendix S2). At baseline, women whose previous child was delivered at home had visited the health facility an average of 4.1 times in the past year and 56.6% had more than three ANC visits; women whose previous child was not delivered at home, or who were primiparous visited the health facility an average of 4.6 times in the past year and 65.7% had more than three ANC visits.

We assessed the effect of the MNH+ intervention among the population of women who had a home delivery immediately prior to the index child. The intervention was associated with a 40% relative increase in facility delivery (RR: 1.42, 95% CI: 1.17, 1.71). Adjusting for covariates resulted in an adjusted RR of 1.43 and 95% CI of 1.14, 1.79 (Table 2).

Within the full study population, the intervention led to the equivalent of an average of 0.5 additional ANC services received out of nine measured (RR: 1.65, 95% CI: 0.99, 2.74). The effect was stronger among the sub‐population of women whose most recent birth was at home (RR: 2.17, 95% CI: 1.23, 3.81). Among the sub‐population of women with their previous birth at home the perception of perceived quality of ANC care was also higher (RR: 1.54, 95% CI: 1.05, 2.27). The intervention led to a reduction in payment for obstetric care among women delivering in their local facility by 3.06 USD (−3.06, 95% CI: −6.04, −0.08). Other assessed measures along the pathway from quality investment to increased utilisation were not affected by the utilisation (Table 3 and Appendix S4).

**Table 3:** Effect of the MNH+ intervention on intermediary outcomes, unadjusted and adjusted difference‐in‐difference analyses

Intermediary outcome	Full study population		Previous home birth	
	RR (95% CI)	Adjusted RR (95% CI)	RR (95% CI)	Adjusted RR (95% CI)
ANC quality improves				
Content of ANC care (index of 9 items,^a^ mean (SD))	1.65 [0.99–2.74]	1.64 [1.00–2.71]	2.17 [1.23–3.81]	2.31 [1.44–3.71]
Perceived quality of ANC care	1.12 [0.87–1.46]	1.14 [0.88–1.47]	1.54 [1.05–2.27]	1.57 [1.07–2.31]
Facility‐community link improves				
Heard of a quality improvement program in local facility	1.11 [0.30–4.10]	1.06 [0.26–4.32]	1.23 [0.39–3.90]	1.14 [0.36–3.64]
Perceived obstetric quality improves				
Perceived quality of delivery care at local MNH+ facility	1.13 [0.79–1.61]	1.13 [0.79–1.62]	1.13 [0.79–1.60]	1.12 [0.78–1.59]
Reduced payment for obstetric care				
Payment for care at local facility (USD), mean (SD)	−3.06 [−6.04 to −0.08]	−3.76 [−7.02 to −0.49]	−1.48 [−3.81 to 0.85]	−2.24 [−4.76 to 0.28]

The predictor of interest is the ‘effect of MNH+’, which is the interaction between time (year dummy variable) and intervention status. We used generalised estimating equations with an exchangeable correlation structure and a log link to estimate risk ratios. This accounts for clustering at the facility level. District is included in both the unadjusted and adjusted models to account for the study design: facilities were stratified by district prior to randomisation. Previous home birth refers to the birth immediately prior to the index child in women who reported two or more births.

a The index of antenatal care includes: weight measured, height measured, blood pressure measured, urine sample collected, blood sample collected, tetanus injection administered, iron supplements provided, antimalarial medications provided and counselled on pregnancy complications.

## Discussion

The MNH+ health system quality improvement intervention resulted in a modest increase in overall facility utilisation. The increased relative risk of 10% corresponds to an absolute increase in utilisation of 6.7 percentage points (95% CI: 0.06, 12.8). The effect of the intervention was stronger among the cohort of women who had delivered their last child at home, among whom the intervention led to an absolute increase in facility delivery of 18.3 percentage points (95% CI: 10.1, 26.6).

This increased utilisation was likely a response to visible efforts to improve the health system (Appendix S7). Past research has shown that one pathway through which facility utilisation for delivery may increase is through better antenatal care (ANC) 32. While improvements in ANC was not the primary focus of this intervention, the equipment and medications supported by the intervention (e.g. blood pressure cuffs and iron tablets), as well as the mentoring and support delivered could have led to improvements in ANC. Our results demonstrate that particularly among the population of women with past home delivery, the intervention led to an improvement in the number of ANC services received. In addition, we hypothesise that seeing the system investment in quality led to an increased interest in the system, spreading confidence in the system as a whole. This is in line with results seen at baseline, which demonstrated that women were less likely to bypass their local clinic if it had received a renovation in the previous year 20.

An increase in utilisation after seeing quality improvement efforts is also consistent with the ‘active patient’ model, where communities alter their behaviours based on signals of improvement in the health system and communication of this to communities 21. A recent evaluation of a maternal and newborn health quality improvement program in Zambia (‘Saving Mothers Giving Life’) found a similar positive effect on facility utilisation for childbirth 33. They posit that the key activities involved in the intervention included community‐based volunteers who were meant to promote facility deliveries and birth preparedness, similar to the ‘peer mamas’ in the MNH+ intervention. These volunteers may be one successful way to link supply‐side quality improvement efforts to the communities they are meant to benefit 33, 34, 35.

It is important to note that the increased utilisation caused by the intervention cannot improve health outcomes without a concurrent improvement in obstetric healthcare quality at the facilities where women are delivering. An evaluation of the effect of the MNH+ intervention on quality in the study facilities found that obstetric quality did not improve. Furthermore, we did not find a difference in women's perception of obstetric care quality in this study. This may in part explain our finding that a large portion of the increased utilisation in intervention facilities occurred in higher‐level facilities (29% of deliveries at baseline compared to 38% at endline occurred in either health centres or hospitals), which are likely to offer higher quality and more comprehensive obstetric care than primary care clinics 11.

The finding that some of the increase in facility utilisation was seen at higher‐level health facilities is consistent with several additional hypotheses. First, the QI intervention may have led providers to recognise the limitations of delivery care provided at the primary care level and encouraged an increase in referrals to health centres and hospitals. Second, the investment in QI combined with the peer outreach program extoling the importance of facility care may have successfully increased women's motivation to access the highest quality of care that they are able to, which is likely at higher‐level facilities. Third, the focus on quality may have led community members to recognise that even with the QI activities, the quality of care on offer at their local clinic was still inferior to that available at higher‐level facilities.

This is one of the first studies to look at the effect of a facility quality intervention on women who have delivered at home in the past. While this was not a pre‐specified focus of the research, given the background of overall high facility utilisation, this group emerged as an important subpopulation that is at greater risk for home delivery and potentially poor outcomes. For example, a recent study in 39 LMIC found that among women who had their first delivery at home, fewer than 12% switched to a facility for the second delivery 29. In our study population, the effect of the QI intervention was much stronger among these women, suggesting that their choice to remain home in the past may have been due to lack of high‐quality facility options nearby. It is noticeable that the intervention did not have an effect on women whose prior delivery was at a health facility. This finding would be unsurprising if the default for these women was to deliver in a health facility – in the same study discussed above, nearly 80% of women who previously delivered in a facility used a facility for their second delivery 29.

There was evidence of increasing facility delivery in this region over time, even in absence of the intervention: among women living in control catchment areas, facility utilisation increased from 72.3% in 2012 to 81.1% in 2016. Similar trends toward increasing facility utilisation for childbirth can be seen in the DHS surveys from 2004–05, 2010, and 2015–16 36, 37, 38. In Pwani Region, some of the trend toward increased utilisation of health facilities may have been due to region‐wide quality improvement efforts, such as the pay for performance program that began in 2011 39. However, on top of these underlying trends, MNH+ had an additional positive affect on facility utilisation. Given the plethora of quality improvement projects occurring in Tanzania, this study benefited from clearly defined and randomly chosen controls in order to prevent major confounding from other, ongoing projects.

This study had several limitations. First, with only 12 clusters in each study arm there was potential for imbalance of unmeasured confounders between intervention and control groups. However, measured variables were similar between the two arms at baseline and there was no evidence that the parallel trends assumption was violated. Second, women in the control areas could have delivered in the treatment facilities, thus increasing the facility utilisation in control areas. However, we found that this occurred rarely: in only 2.7% of women at baseline and 1.8% at endline. This may be due to large (on average more than 10 kilometers) distance between the nearest study facilities. Third, the 24 health facilities included in this study were not chosen at random. They represent the facilities with the most deliveries in their district. Because it is unlikely that the facilities with fewer deliveries were higher functioning at baseline than the selected facilities, we do not expect that our results would have changed if implemented in these less busy facilities. Finally, the potential mechanisms of effect, including the quality of antenatal care received were measured through maternal report, allowing for potential recall bias. If women in control facilities were more likely to misreport an increased receipt in ANC services, then this could have biased this finding.

This study provides evidence that health systems improvement efforts can potentially motivate women, especially past non‐users, to use health facilities for delivery. Future health system interventions should continue to focus on and evaluate quality, as this focus has potential to both increase quality and utilisation, which in turn can lead to improved population health.

## Trial registration

ISRCTN 17107760: http://www.isrctn.com/ISRCTN17107760

## Funding

NIH R01AI093182. No funding bodies had any role in study design, data collection and analysis, decision to publish, or preparation of the manuscript.

## Supporting information


**Appendix S1.** Study eligibility and analysis flow diagram.
**Appendix S2.** Descriptive statistics of the sub‐group of women with a previous home delivery, Pwani region, Tanzania (2011–2012 and 2015–2016).
**Appendix S3.** Effect of MNH+ intervention on facility utilisation for childbirth, unadjusted and adjusted difference‐in‐difference analyses stratified by risk level.
**Appendix S4.** Effect of the MNH+ intervention on intermediary outcomes (full study population).
**Appendix S5.** Methods and results of sensitivity analyses.
**Appendix S6.** Changes in place of delivery from baseline to endline by intervention status.
**Appendix S7.** Example of the delivery room in a study facility before (A) and after (B) quality improvement activities.

## References

[1] MDG Report 2015: Assessing progress in Africa toward the Millennium Development Goals. Addis Ababa, Ethiopia: United Nations Economic Commission for Africa, 2015.

[2] Montagu D , Sudhinaraset M , Diamond‐Smith N *et al* Where women go to deliver: understanding the changing landscape of childbirth in Africa and Asia. Health Policy Plan 2017: 32: 1146–1152. 28541422 10.1093/heapol/czx060 PMC5886217

[3] Koblinsky M , Moyer CA , Calvert C *et al* Quality maternity care for every woman, everywhere: a call to action. Lancet 2016: 388: 2307–2320. 27642018 10.1016/S0140-6736(16)31333-2

[4] Lee AC , Cousens S , Darmstadt GL *et al* Care during labor and birth for the prevention of intrapartum‐related neonatal deaths: a systematic review and Delphi estimation of mortality effect. BMC Public Health 2011: 11(Suppl 3): S10. 10.1186/1471-2458-11-S3-S10 PMC3231883 21501427

[5] Moyer CA , Mustafa A . Drivers and deterrents of facility delivery in sub‐Saharan Africa: a systematic review. Reprod Health 2013: 10: 40. 23962135 10.1186/1742-4755-10-40 PMC3751820

[6] McKinnon B , Harper S , Kaufman JS , Bergevin Y . Removing user fees for facility‐based delivery services: a difference‐in‐differences evaluation from ten sub‐Saharan African countries. Health Policy Plan 2015: 30: 432–441. 24816570 10.1093/heapol/czu027 PMC4385820

[7] Nyamtema AS , Urassa DP , van Roosmalen J . Maternal health interventions in resource limited countries: a systematic review of packages, impacts and factors for change. BMC Pregnancy Childbirth 2011: 11: 30. 21496315 10.1186/1471-2393-11-30 PMC3090370

[8] Hunter BM , Murray SF . Demand‐side financing for maternal and newborn health: what do we know about factors that affect implementation of cash transfers and voucher programmes? BMC Pregnancy Childbirth 2017: 17: 262. 28854877 10.1186/s12884-017-1445-y PMC5577737

[9] Audo MO , Ferguson A , Njoroge PK . Quality of health care and its effects in the utilisation of maternal and child health services in Kenya. East Afr Med J 2005: 82: 547–553. 16463747 10.4314/eamj.v82i11.9407

[10] Gage AD , Leslie HH , Bitton A *et al* Does quality influence utilization of primary health care? Evidence from Haiti Glob Health 2018: 14: 59. 10.1186/s12992-018-0379-0 PMC6011404 29925416

[11] Kruk ME , Leslie HH , Verguet S , Mbaruku GM , Adanu RM , Langer A . Quality of basic maternal care functions in health facilities of five African countries: an analysis of national health system surveys. Lancet Glob Health 2016: 4: e845–e855. 27670090 10.1016/S2214-109X(16)30180-2

[12] Larson E , Vail D , Mbaruku GM , Mbatia R , Kruk ME . Beyond utilization: measuring effective coverage of obstetric care along the quality cascade. Int J Qual Health Care 2017: 29: 104–110. 27920246 10.1093/intqhc/mzw141 PMC5890864

[13] Nesbitt RC , Lohela TJ , Manu A *et al* Quality along the Continuum: a health facility assessment of intrapartum and postnatal care in Ghana. PLoS ONE 2013: 8: e81089. 24312265 10.1371/journal.pone.0081089 PMC3842335

[14] Sharma J , Leslie HH , Kundu F , Kruk ME . Poor Quality for Poor Women? Inequities in the Quality of Antenatal and Delivery Care in Kenya. PLoS ONE 2017: 12: e0171236. 28141840 10.1371/journal.pone.0171236 PMC5283741

[15] Hanson K , McPake B , Nakamba P , Archard L . Preferences for hospital quality in Zambia: results from a discrete choice experiment. Health Econ 2005: 14: 687–701. 15619273 10.1002/hec.959

[16] Kruk ME , Rockers PC , Tornorlah Varpilah S , Macauley R . Population preferences for health care in liberia: insights for rebuilding a health system. Health Serv Res 2011: 46: 2057–2078. 21517835 10.1111/j.1475-6773.2011.01266.x PMC3392998

[17] Larson E , Vail D , Mbaruku GM , Kimweri A , Freedman LP , Kruk ME . Moving toward patient‐centered care in Africa: a discrete choice experiment of preferences for delivery care among 3,003 Tanzanian women. PLoS ONE 2015: 10: e0135621. 26262840 10.1371/journal.pone.0135621 PMC4532509

[18] Larson E , Hermosilla S , Kimweri A , Mbaruku GM , Kruk ME . Determinants of perceived quality of obstetric care in rural Tanzania: a cross‐sectional study. BMC Health Serv Res 2014: 14: 483. 25326007 10.1186/1472-6963-14-483 PMC4283093

[19] Karkee R , Lee AH , Binns CW . Bypassing birth centres for childbirth: an analysis of data from a community‐based prospective cohort study in Nepal. Health Policy Plan 2013: 30: 1–7. 24270520 10.1093/heapol/czt090

[20] Kruk ME , Hermosilla S , Larson E , Mbaruku GM . Bypassing primary care clinics for childbirth: a cross‐sectional study in the Pwani region, United Republic of Tanzania. Bull World Health Organ 2014: 92: 246–253. 24700992 10.2471/BLT.13.126417 PMC3967574

[21] Leonard KL . Active patients in rural African health care: implications for research and policy. Health Policy Plan 2013: 29: 85–95. 23307907 10.1093/heapol/czs137

[22] Kruk ME , Vail D , Austin‐Evelyn K *et al* Evaluation Of A Maternal Health Program In Uganda And Zambia Finds Mixed Results On Quality Of Care And Satisfaction. Health Aff 2016: 35: 510–519. 10.1377/hlthaff.2015.0902 26953307

[23] Rowe AK , de Savigny D , Lanata CF , Victora CG . How can we achieve and maintain high‐quality performance of health workers in low‐resource settings? Lancet 2005: 366: 1026–1035. 16168785 10.1016/S0140-6736(05)67028-6

[24] Semrau KEA , Hirschhorn LR , Marx Delaney M *et al* Outcomes of a Coaching‐Based WHO Safe Childbirth Checklist Program in India. N Engl J Med 2017: 377: 2313–2324. 29236628 10.1056/NEJMoa1701075 PMC5672590

[25] Andersen RM . Revisiting the behavioral model and access to medical care: does it matter? J Health Soc Behav 1995: 36: 1–10. 7738325

[26] Kruk ME , Hermosilla S , Larson E *et al* Who is left behind on the road to universal facility delivery? A cross‐sectional multilevel analysis in rural Tanzania. TMIH 2015: 20: 1057–1066. 10.1111/tmi.12518 PMC4490971 25877211

[27] Filmer D , Pritchett LH . Estimating wealth effects without expenditure data ‐ Or tears: an application to educational enrollments in states of India. Demography 2001: 38: 115–132. 11227840 10.1353/dem.2001.0003

[28] Zeger SL , Liang KY . Longitudinal data analysis for discrete and continuous outcomes. Biometrics 1986: 42: 121–130. 3719049

[29] Benova L , Macleod D , Radovich E , Lynch CA , Campbell OMR . Should I stay or should I go?: consistency and switching of delivery locations among new mothers in 39 Sub‐Saharan African and South/Southeast Asian countries. Health Policy Plan 2017: 32: 1294–1308. 28981668 10.1093/heapol/czx087 PMC5886240

[30] Hotchkiss DR , Piccinino L , Malaj A , Berruti AA , Bose S . Addressing the phenomenon of bypassing in Albania: the impact of a primary health care strengthening intervention. Int J Health Plann Manage 2007: 22: 225–243. 17624878 10.1002/hpm.869

[31] Rockers PC , Kruk ME , Laugesen MJ . Perceptions of the health system and public trust in government in low‐ and middle‐income countries: evidence from the World Health Surveys. J Health Polit Policy Law 2012: 37: 405–437. 22323234 10.1215/03616878-1573076

[32] Rockers PC , Wilson ML , Mbaruku G , Kruk ME . Source of antenatal care influences facility delivery in rural Tanzania: a population‐based study. Matern Child Health J 2009: 13: 879–885. 18810618 10.1007/s10995-008-0412-7

[33] Henry EG , Thea DM , Hamer DH *et al* The impact of a multi‐level maternal health programme on facility delivery and capacity for emergency obstetric care in Zambia. Glob Public Health 2018: 13: 1481–1494. 28994352 10.1080/17441692.2017.1385824 PMC6176772

[34] Lee ACC , Lawn JE , Cousens S *et al* Linking families and facilities for care at birth: what works to avert intrapartum‐related deaths? Int J Gynaecol Obstet 2009: 107(Suppl): S65–S88. 19815201 10.1016/j.ijgo.2009.07.012 PMC3428847

[35] Ensor T , Green C , Quigley P , Badru AR , Kaluba D , Kureya T . Mobilizing communities to improve maternal health: results of an intervention in rural Zambia. Bull World Health Organ 2014: 92: 51–59. 24391300 10.2471/BLT.13.122721 PMC3865550

[36] Tanzania Demographic and Health Survey 2004–05. Dar es Salaam, Tanzania: National Bureau of Statistics (NBS) [Tanzania] and ORC Macro, 2005.

[37] Tanzania Demographic and Health Survey and Malaria Indicator Survey 2015–2016 Final Report. Dar es Salaam, Tanzania and Rockville, Maryland, USA: Ministry of Health, Community Development, Gender, Elderly and Children (MoHCDGEC) [Tanzania Mainland], Ministry of Health (MoH) [Zanzibar], National Bureau of Statistics (NBS), Office of the Chief Government Statistician (OCGS), and ICF, 2016.

[38] DHS . Tanzania Demographic and Health Survey 2010. Dar es Salaam: National Bureau of Statistics (NBS) [Tanzania]; ICF Macro, 2011.

[39] Binyaruka P , Patouillard E , Powell‐Jackson T , Greco G , Maestad O , Borghi J . Effect of paying for performance on utilisation, quality, and user costs of health services in Tanzania: a controlled before and after study. PLoS ONE 2015: 10: e0135013. 26317510 10.1371/journal.pone.0135013 PMC4552688

