# Supplementary material for: Effect of a maternal and newborn health system quality improvement project on the use of facilities for childbirth: a cluster‐randomised study in rural Tanzania
Source: Trop Med Int Health. 2019 Mar 11;24(5):636–46. doi: 10.1111/tmi.13220 (PMC6499631; doi:10.1111/tmi.13220)

**Appendix Figure A1.** Study eligibility and analysis flow diagram.


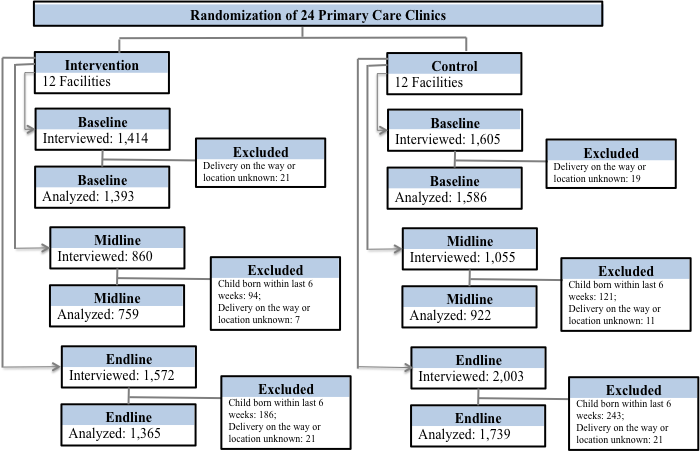


**Appendix Table A2.** Descriptive statistics of the sub-group of women with a previous home delivery, Pwani region, Tanzania (2011-2012 and 2015-2016)

|  | Baseline | | Endline | |
| --- | --- | --- | --- | --- |
|  | Control (N=455) | Treatment (N=382) | Control (N=306) | Treatment (N=232) |
| Demographics |  |  |  |  |
| Age (mean) | 29.7 | 29.7 | 30.6 | 30.6 |
| Education (categorical) |  |  |  |  |
| No formal | 40% | 38% | 34% | 41% |
| Some primary | 14% | 16% | 17% | 10% |
| Completed primary | 44% | 44% | 45% | 47% |
| Any secondary | 2% | 3% | 3% | 3% |
| Farmer or homemaker | 92% | 88% | 89% | 86% |
| Muslim | 80% | 83% | 74% | 80% |
| Married or living with partner | 89% | 86% | 88% | 91% |
| Household assets |  |  |  |  |
| Media index (mean)^1^ | 2.97 | 2.74 | 2.55 | 2.63 |
| Household wealth: richest 20%^2^ | 12% | 11% | 9% | 11% |
| Mobile phone | 66% | 65% | 87% | 85% |
| Electricity | 4% | 2% | 13% | 14% |
| Consumes >2 meals per day | 86% | 88% | 87% | 82% |
| Delivery characteristics |  |  |  |  |
| Birth during harvest season | 13% | 15% | 18% | 18% |
| Delivery at facility^3^ | 43% | 43% | 43% | 60% |
| Community characteristics |  |  |  |  |
| Village has paved road | 28% | 39% | 43% | 45% |
| District |  |  |  |  |
| Bagamoyo | 50% | 41% | 60% | 60% |
| Kibaha Rural | 11% | 14% | 9% | 8% |
| Kisarawe | 19% | 28% | 19% | 19% |
| Mkuranga | 20% | 18% | 12% | 12% |

Notes: Previous home birth refers to the birth immediately prior to the index child in women who reported two or more births.

^1^ Media index range (0,12)

^2^ Wealth index constructed using baseline asset weights for both baseline and endline cohorts

^3^ Dependent variable

**Appendix Table A3.1.** Effect of MNH+ intervention on facility utilization for childbirth, unadjusted and adjusted difference-in-difference analyses stratified by risk level

|  | Control Baseline  N (%) | Control  Endline  N (%) | Intervention Baseline  N (%) | Intervention Endline  N (%) | RR (95% CI) | Fisher permutation test p-value | adjusted  RR (95% CI) |
| --- | --- | --- | --- | --- | --- | --- | --- |
| Full population | 1,146 (72.3) | 1,411 (81.1) | 999 (71.7) | 1,165 (85.3) | 1.10 (1.00, 1.21) | 0.118 | 1.08 (0.98, 1.19) |
| Last birth at home | 195 (42.9) | 130 (42.5) | 163 (42.7) | 140 (60.3) | 1.42 (1.17, 1.71) | 0.020 | 1.41 (1.13, 1.77) |
| Primiparous or last birth in facility | 944 (84.1) | 1,277 (89.5) | 831 (82.6) | 1,017 (90.6) | 1.05 (0.98, 1.12) | 0.294 | 1.04 (0.97, 1.11) |

Notes: “RR” refers to the risk ratio, calculated using generalized estimating equations with an exchangeable correlation structure and a log link to estimate risk ratios. The Fisher permutation test p-value gives the p-value determined using 50. All models are adjusted for district; the “adjusted” model is additionally adjusted for the covariates listed in exhibit 3.

**Appendix Table A4.1a** Effect of the MNH+ intervention on intermediary outcomes (full study population)

| Intermediary outcome | Control Baseline  N (%) | Control Endline  N (%) | Intervention Baseline  N (%) | Intervention Endline  N (%) | RR (95% CI) | adjusted RR (95% CI) |
| --- | --- | --- | --- | --- | --- | --- |
| Referred to this facility ^a^ | - | 278 (19.7) | - | 266 (22.9) | 1.06 [0.81 - 1.39] | 1.08 [0.83 - 1.43] |
| Content of ANC care (index of 9 items, mean (SD)) | 6.8 (1.6) | 7.0 (1.7) | 6.8 (1.6) | 7.4 (1.3) | 1.65 [0.99 - 2.74] | 1.64 [1.00 - 2.71] |
| Weight measured | 954 (91) | 1013 (92.3) | 964 (96) | 1013 (99.6) | 1.02 [0.88 - 1.17] | 1.02 [0.89 - 1.17] |
| Height measured | 775 (74.7) | 742 (67.6) | 804 (80.6) | 799 (78.6) | 1.10 [0.93 - 1.30] | 1.11 [0.94 - 1.32] |
| BP measured | 846 (81) | 875 (79.8) | 844 (84.1) | 924 (90.9) | 1.12 [0.96 - 1.30] | 1.12 [0.97 - 1.30] |
| Urine sample collected | 489 (46.8) | 621 (56.6) | 435 (43.4) | 557 (54.8) | 1.11 [0.87 - 1.41] | 1.11 [0.87 - 1.42] |
| Blood sample collected | 987 (94.2) | 864 (78.8) | 937 (93.3) | 799 (78.6) | 1.02 [0.90 - 1.16] | 1.01 [0.89 - 1.15] |
| Tetanus injection administered | 814 (77.7) | 790 (72.1) | 766 (76.3) | 804 (79.3) | 1.12 [0.97 - 1.29] | 1.12 [0.97 - 1.28] |
| Iron supplements provided | 766 (73.2) | 1063 (96.9) | 734 (73.1) | 996 (97.9) | 1.01 [0.86 - 1.18] | 1.00 [0.85 - 1.17] |
| Antimalarial meds provided | 840 (80.2) | 999 (91.2) | 721 (72.2) | 929 (91.5) | 1.09 [0.95 - 1.26] | 1.10 [0.95 - 1.26] |
| Counseled on pregnancy complications | 693 (66.3) | 692 (63.7) | 621 (62) | 719 (71.5) | 1.19* [1.02 - 1.38] | 1.18* [1.02 - 1.37] |
| Perceived quality of ANC care | 329 (31.5) | 489 (44.7) | 356 (35.5) | 563 (55.4) | 1.12 [0.87 - 1.46] | 1.14 [0.88 - 1.47] |
| Satisfaction with ANC care | 438 (45.5) | 667 (60.8) | 447 (49.3) | 656 (64.6) | 1.01 [0.70 - 1.47] | 1.02 [0.70 - 1.49] |
| Heard of a quality improvement program in local facility | 129 (8) | 102 (5.8) | 196 (13.9) | 179 (12.9) | 1.11 [0.30 - 4.10] | 1.06 [0.26 - 4.32] |
| Perceived quality of delivery care at local MNH+ facility: Community rating | 3.3 (0.4) | 3.5 (0.4) | 3.3 (0.5) | 3.7 (03) | 1.13 [0.79 - 1.61] | 1.13 [0.79 - 1.62] |
| Satisfaction with overall health system | 560 (35.4) | 863 (49.1) | 441 (31.6) | 606 (43.8) | 0.99 [0.66 - 1.50] | 1.00 [0.67 - 1.49] |
| Trust health system authorities to care about patient opinions | 410 (26.1) | 787 (45.6) | 313 (22.7) | 563 (41.1) | 1.01 [0.68 - 1.48] | 0.98 [0.67 - 1.45] |
| Payment for care at local facility (USD), mean (SD) | 6.02 (5.00) | 13.39 (19.96) | 5.79 (4.49) | 10.04 (6.94) | -3.06* [-6.04 - -0.08] | -3.76* [-7.02 - -0.49] |

Notes for tables 3.1a-c:

^a^ Referral only assessed at endline

Each row represents a distinct model with the listed intermediary outcome as the dependent variable. The reported risk ratios show the effect of the intervention (which is the interaction between time (year dummy variable) and intervention status) on the intermediary outcome. We used generalized estimating equations with an exchangeable correlation structure and a log link to estimate risk ratios in the case of binary outcomes and the identity link for “content of ANC care” and “cost of care at local facility”. The adjusted models are adjusted for the covariates listed in exhibit 3.

* p <0.05; ** p<0.01; *** p<0.001

**Appendix Table A4.1b** Effect of the MNH+ intervention on intermediary outcomes (last birth at home)

| Intermediary outcome | Control Baseline  N (%) | Control Endline  N (%) | Intervention Baseline  N (%) | Intervention Endline  N (%) | RR (95% CI) | adjusted RR (95% CI) |
| --- | --- | --- | --- | --- | --- | --- |
| Referred to this facility^a^ | - | 18 (14) | - | 25 (17.9) | 1.29 [0.71 - 2.34] | 1.23 [0.73 - 2.08] |
| Content of ANC care (index of 9 items, mean (SD)) | 6.7 (1.7) | 6.4 (1.7) | 6.6 (1.6) | 7.1 (1.4) | 2.17** [1.23 - 3.81] | 2.31*** [1.44 - 3.71] |
| Weight measured | 298 (89.2) | 206 (91.6) | 280 (96.2) | 189 (99) | 0.98 [0.79 - 1.21] | 1.00 [0.86 - 1.16] |
| Height measured | 243 (73) | 133 (59.1) | 222 (77.1) | 148 (77.5) | 1.18 [0.97 - 1.43] | 1.18 [0.98 - 1.43] |
| BP measured | 263 (79.2) | 162 (72) | 233 (80.3) | 163 (85.3) | 1.18* [1.00 - 1.38] | 1.20* [1.04 - 1.39] |
| Urine sample collected | 131 (39.2) | 104 (46.2) | 108 (37.4) | 88 (46.1) | 1.07 [0.73 - 1.56] | 1.10 [0.76 - 1.59] |
| Blood sample collected | 316 (94.6) | 147 (65.3) | 270 (93.1) | 134 (70.2) | 1.08 [0.88 - 1.31] | 1.08 [0.90 - 1.30] |
| Tetanus injection administered | 261 (78.4) | 135 (60.3) | 196 (67.4) | 138 (72.6) | 1.47*** [1.30 - 1.65] | 1.45*** [1.28 - 1.64] |
| Iron supplements provided | 239 (71.8) | 216 (96) | 210 (72.2) | 184 (96.3) | 0.97 [0.82 - 1.14] | 0.94 [0.79 - 1.12] |
| Antimalarial meds provided | 264 (79) | 201 (89.3) | 203 (70.5) | 180 (94.2) | 1.14 [0.91 - 1.43] | 1.15 [0.92 - 1.44] |
| Counseled on pregnancy complications | 208 (62.3) | 132 (59.5) | 173 (60.1) | 129 (68.6) | 1.24* [1.02 - 1.50] | 1.25* [1.03 - 1.51] |
| Perceived quality of ANC care | 110 (33.1) | 86 (38.2) | 93 (32.1) | 105 (55) | 1.54* [1.05 - 2.27] | 1.57* [1.07 - 2.31] |
| Satisfaction with ANC care | 141 (43.5) | 135 (60) | 126 (45.8) | 122 (63.9) | 1.09 [0.70 - 1.71] | 1.14 [0.76 - 1.71] |
| Heard of a quality improvement program in local facility | 31 (6.7) | 20 (6.4) | 40 (10.4) | 32 (13.3) | 1.23 [0.39 - 3.90] | 1.14 [0.36 - 3.64] |
| Perceived quality of delivery care at local MNH+ facility: Community rating | 3.2 (0.4) | 3.5 (0.5) | 3.2 (0.5) | 3.7 (0.4) | 1.13 [0.79 - 1.60] | 1.12 [0.78 - 1.59] |
| Satisfaction with overall health system | 161 (35.5) | 157 (50.3) | 124 (32.9) | 124 (51.5) | 1.13 [0.66 - 1.94] | 1.10 [0.66 - 1.82] |
| Trust health system authorities to care about patient opinions | 128 (28.5) | 137 (44.3) | 99 (26.8) | 117 (48.5) | 1.19 [0.74 - 1.91] | 1.09 [0.66 - 1.77] |
| Payment for care at local facility (USD) | 6.18 (6.30) | 11.20 (3.77) | 6.33 (5.10) | 8.86 (4.49) | -1.48 [-3.81 - 0.85] | -2.24 [-4.76 - 0.28] |

**Appendix Table A4.1c** Effect of the MNH+ intervention on intermediary outcomes (primiparous or last birth at facility)

| Intermediary outcome | Control Baseline  N (%) | Control Endline  N (%) | Intervention Baseline  N (%) | Intervention Endline  N (%) | RR (95% CI) | adjusted RR (95% CI) |
| --- | --- | --- | --- | --- | --- | --- |
| Referred to this facility^a^ | - | 260 (20.4) | - | 240 (23.6) | 1.03 [0.78 - 1.35] | 1.05 [0.80 - 1.38] |
| Content of ANC care (index of 9 items, mean (SD)) | 6.9 (1.6) | 7.1 (1.6) | 6.9 (1.6) | 7.5 (1.3) | 1.50 [0.89 - 2.52] | 1.46 [0.86 - 2.47] |
| Weight measured | 652 (92) | 868 (100) | 681 (95.9) | 818 (100) | 1.03 [0.90 - 1.18] | 1.03 [0.90 - 1.18] |
| Height measured | 528 (75.4) | 868 (100) | 579 (81.9) | 818 (100) | 1.08 [0.92 - 1.27] | 1.09 [0.92 - 1.29] |
| BP measured | 578 (81.8) | 868 (100) | 608 (85.6) | 818 (100) | 1.10 [0.94 - 1.29] | 1.10 [0.94 - 1.28] |
| Urine sample collected | 357 (50.6) | 868 (100) | 325 (45.7) | 818 (100) | 1.12 [0.90 - 1.38] | 1.11 [0.88 - 1.40] |
| Blood sample collected | 666 (93.9) | 868 (100) | 664 (93.4) | 818 (100) | 1.00 [0.89 - 1.12] | 0.99 [0.88 - 1.12] |
| Tetanus injection administered | 550 (77.6) | 867 (100) | 567 (79.9) | 816 (100) | 1.04 [0.89 - 1.22] | 1.03 [0.89 - 1.20] |
| Iron supplements provided | 523 (73.9) | 868 (100) | 522 (73.5) | 818 (100) | 1.02 [0.87 - 1.18] | 1.01 [0.87 - 1.18] |
| Antimalarial meds provided | 571 (80.6) | 866 (100) | 515 (72.8) | 816 (100) | 1.08 [0.94 - 1.24] | 1.08 [0.95 - 1.23] |
| Counseled on pregnancy complications | 480 (67.9) | 861 (100) | 445 (62.7) | 811 (100) | 1.17* [1.00 - 1.38] | 1.16 [0.99 - 1.37] |
| Perceived quality of ANC care | 218 (30.8) | 866 (100) | 261 (36.8) | 817 (100) | 1.01 [0.78 - 1.30] | 1.01 [0.79 - 1.30] |
| Satisfaction with ANC care | 294 (46.4) | 868 (100) | 319 (50.7) | 816 (100) | 0.98 [0.69 - 1.40] | 0.99 [0.69 - 1.41] |
| Heard of a quality improvement program in local facility | 96 (8.5) | 80 (5.6) | 156 (15.3) | 147 (13) | 1.09 [0.31 - 3.86] | 1.03 [0.25 - 4.13] |
| Perceived quality of delivery care at local MNH+ facility: Community rating | 3.3 (0.4) | 3.5 (0.4) | 3.3 (0.5) | 3.7 (0.3) | 1.12 [0.77 - 1.61] | 1.11 [0.76 - 1.61] |
| Satisfaction with overall health system | 396 (35.4) | 700 (48.7) | 316 (31.3) | 478 (42.3) | 0.97 [0.66 - 1.42] | 0.98 [0.67 - 1.42] |
| Trust health system authorities to care about patient opinions | 279 (25) | 647 (46) | 212 (21.2) | 444 (39.6) | 0.98 [0.67 - 1.42] | 0.97 [0.66 - 1.42] |
| Payment for care at local facility (USD) | 5.96 (4.62) | 13.64 (21.07) | 5.68 (4.35) | 10.26 (7.24) | -3.09 [-6.35 - 0.16] | -3.85* [-7.38 - -0.32] |

**Appendix Table A4.2** Associations between client-reported intermediary outcomes and facility utilization for childbirth at baseline

|  | Full sample | Last birth at home | Primiparous or last birth at health facility |
| --- | --- | --- | --- |
| Intermediary outcomes | RR (95% CI) | RR (95% CI) | RR (95% CI) |
| Content of ANC care (index of 9 items, mean (SD)) | 1.04*** [1.02 - 1.06] | 1.10*** [1.05 - 1.14] | 1.02** [1.01 - 1.04] |
| Weight measured | 1.12* [1.02 - 1.23] | 1.97** [1.21 - 3.22] | 1.00 [0.93 - 1.07] |
| Height measured | 1.08* [1.00 - 1.17] | 0.91 [0.79 - 1.05] | 1.13** [1.03 - 1.23] |
| BP measured | 1.12** [1.03 - 1.20] | 1.17 [0.99 - 1.37] | 1.09 [1.00 - 1.18] |
| Urine sample collected | 1.08*** [1.04 - 1.13] | 1.27** [1.10 - 1.48] | 1.01 [0.95 - 1.07] |
| Blood sample collected | 0.99 [0.91 - 1.09] | 0.94 [0.68 - 1.31] | 1.01 [0.91 - 1.11] |
| Tetanus injection administered | 1.04 [0.97 - 1.11] | 1.01 [0.85 - 1.19] | 0.98 [0.92 - 1.04] |
| Iron supplements provided | 1.05 [1.00 - 1.11] | 1.17 [0.98 - 1.40] | 1.03 [0.98 - 1.09] |
| Antimalarial meds provided | 1.11* [1.02 - 1.22] | 1.35** [1.09 - 1.69] | 1.07** [1.02 - 1.12] |
| Counseled on pregnancy complications | 1.11*** [1.08 - 1.15] | 1.27** [1.09 - 1.48] | 1.06** [1.02 - 1.10] |
| Perceived quality of ANC care | 0.99 [0.94 - 1.05] | 1.08 [0.94 - 1.24] | 0.99 [0.94 - 1.03] |
| Satisfaction with ANC care | 1.03 [0.97 - 1.09] | 1.01 [0.85 - 1.20] | 1.02 [0.97 - 1.07] |
| Heard of a quality improvement program in local facility | 1.14*** [1.06 - 1.21] | 1.34** [1.07 - 1.67] | 1.04 [0.99 - 1.09] |
| Perceived quality of delivery care at local MNH+ facility: Community rating | 1.10 [0.94 - 1.28] | 1.03 [0.81 - 1.30] | 1.09 [0.96 - 1.24] |
| Satisfaction with overall health system | 0.95 [0.89 - 1.01] | 0.95 [0.78 - 1.14] | 0.95 [0.90 - 1.01] |
| Trust health system authorities to care about patient opinions | 0.96 [0.90 - 1.01] | 1.03 [0.90 - 1.19] | 0.96 [0.92 - 1.00] |
| Payment for care at local facility (USD); Community average | 0.99 [0.97 - 1.02] | 0.98 [0.93 - 1.03] | 1.00 [0.98 - 1.02] |

Notes: The measures of association in this table are not causal. Each row represents a distinct model with the listed “intermediary outcome” as the independent variable of interest. The dependent variable for each model is facility utilization for childbirth. We used generalized estimating equations with an exchangeable correlation structure and a log link to estimate risk ratios. All models include district as a covariate.

**Appendix 5.** Methods and results of sensitivity analyses.

Methods: We conducted four sensitivity analyses. First, in order for the predictors of utilization to confound the association between the intervention and utilization, they would need to be associated with intervention status. As a robustness check we therefore planned to analyze a model with only confounders that were shown to be associated with the intervention status at baseline. We found none of the above listed confounders were associated with intervention status at baseline, and we therefore present two models in the main paper, the unadjusted and fully adjusted. Second, to account for missing covariate data in the fully adjusted model we conducted a sensitivity check with a multiply imputed dataset. Data were imputed using chained equations with 20 imputations. Third, we used the monthly data to conduct an interrupted time series analysis using the control group as a comparator. Fourth, we conducted a cross-sectional analysis comparing treatment to control groups at endline. Finally, we used a permutation test with 50 repetitions to calculate significance for each model as an alternative way to account for the clustered data structure.([40](#_ENREF_40))

Results: There were 80 observations (1.3%) with missing covariates. When these data were imputed the adjusted relative risk does not qualitatively change: 1.09 with a 95% CI of 0.99, 1.20. In the interrupted time series analysis, there was no evidence of change in trend of utilization between the intervention and control groups. In the post-only analysis, the unadjusted relative risk is 1.02 with a 95% confidence interval of 0.96, 1.08 and the adjusted relative risk is 1.03 with a 95% confidence interval of 0.97, 1.10. Finally, the p-value for the Fischer Permutation test was not significant for the full population (p=0.118), but was significant for women whose last birth was at home (p=0.020).

**Table A5.1.** Results of the interrupted time series analysis to assess the effect of the MNH+ intervention on facility utilization for childbirth, 2011-2016.

|  | RR [95% CI] | p-value |
| --- | --- | --- |
| Time from intervention start | 1.00 [0.99, 1.01] | 0.785 |
| Post-intervention dummy | 1.07 [0.89, 1.29] | 0.465 |
| Time X Post | 1.00 [0.99, 1.01] | 0.918 |
| Intervention dummy | 0.92 [0.73, 1.16] | 0.466 |
| Time X Intervention | 0.99 [0.98, 1.01] | 0.488 |
| Post X Intervention | 1.15 [0.94, 1.40] | 0.185 |
| Time X Post X Intervention | 1.01 [0.99, 1.02] | 0.481 |

Table notes: This table was constructed using the following identification strategy:

$${log(P(Y}_{t}=1)=\beta_{0}+\beta_{1}T+\beta_{2}X_{t}+\beta_{3}TX_{t}+\beta_{4}Z+\beta_{5}ZT+\beta_{6}ZX_{t}+\beta_{7}ZTX_{t}$$

Where $\beta_{1}$ = (Time) time trend; $\beta_{2}$ = (Post-intervention dummy) level change following the intervention; $\beta_{3}$ = (Time X Post) slope change following the intervention; $\beta_{4}$ = (Intervention dummy) difference between intervention and control before the intervention; $\beta_{5}$ = (Time X Intervention) difference between intervention and control in trend before the intervention (checks the parallel trends assumption, should be non-significant and null); $\beta_{6}$= (Post X Intervention) difference between intervention and control immediately after the intervention; $\beta_{7}$ = (Time X Post X Intervention) difference in difference in slopes (main measure of effect). We used generalized estimating equations with an exchangeable correlation structure and a log link to estimate risk ratios.

**Appendix Figure A6.** Changes in place of delivery from baseline to endline by intervention status.

**Appendix Figure A7.** Example of the delivery room in a study facility before (A) and after (B) quality improvement activities.


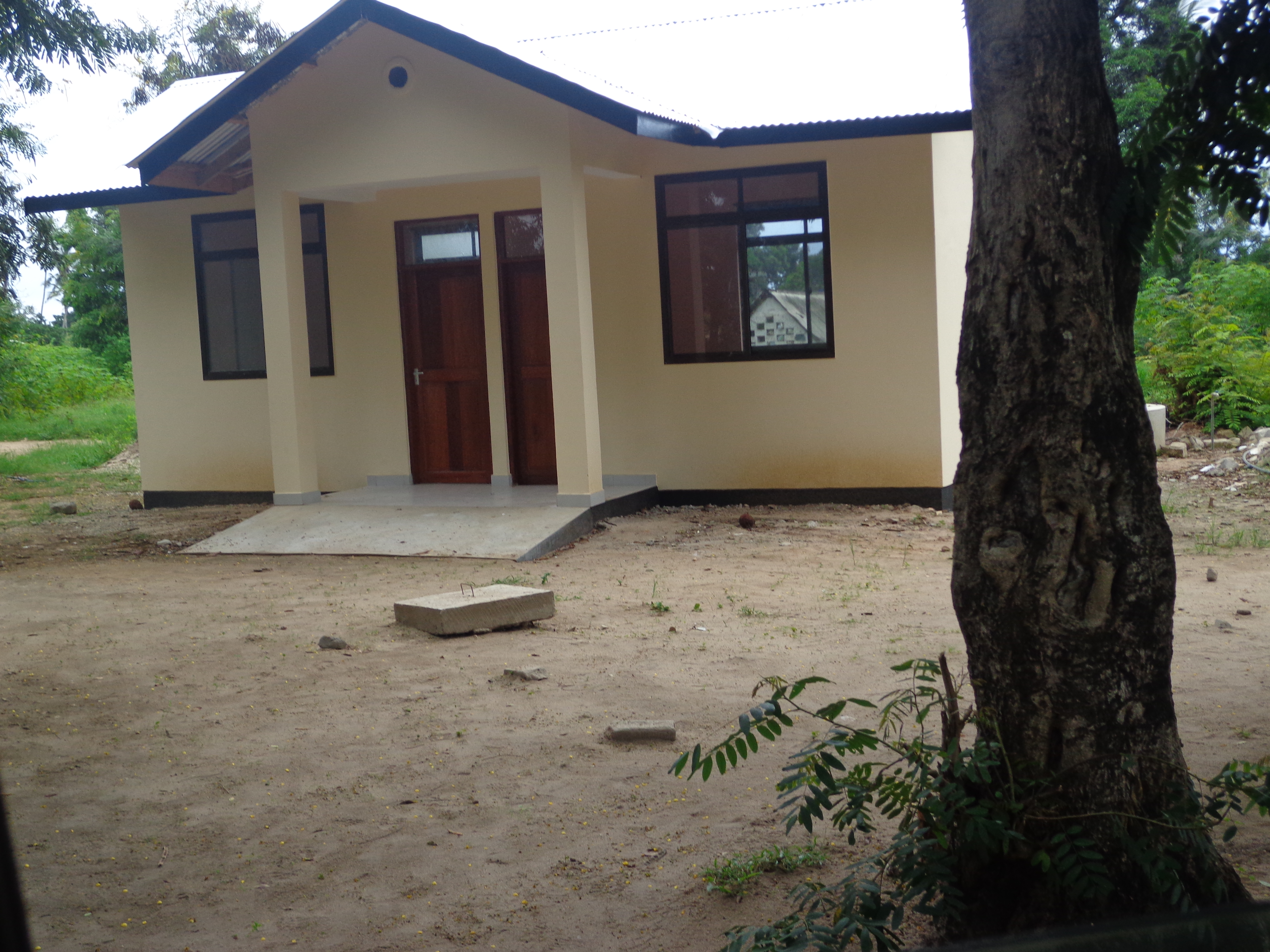
A. B.


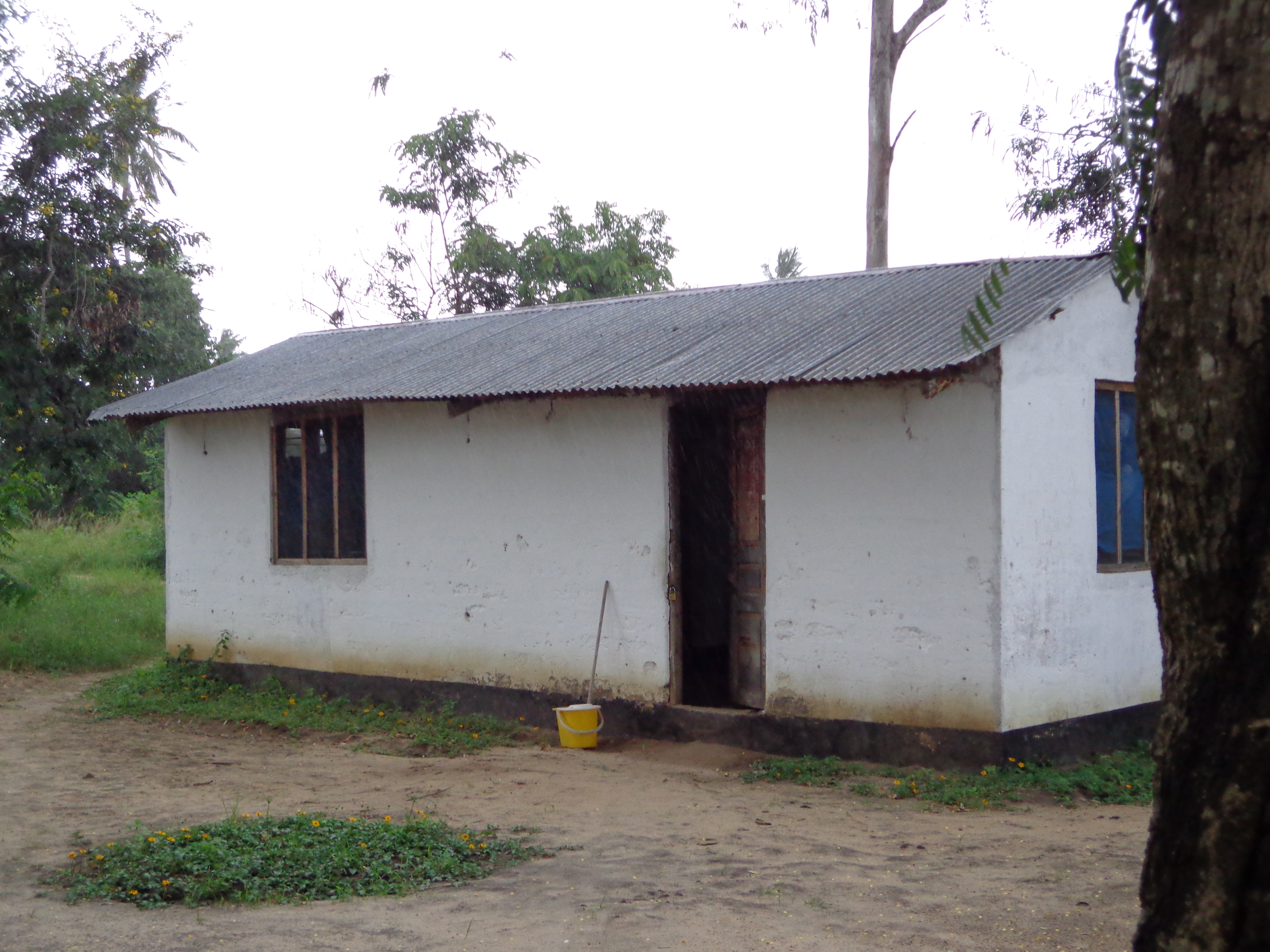

Supplement: Supplementary file 1 — Appendix S1. Study eligibility and analysis flow diagram. Appendix S2. Descriptive statistics of the sub‐group of women with a previous home delivery, Pwani region, Tanzania (2011–2012 and 2015–2016). Appendix S3. Effect of MNH+ intervention on facility utilisation for childbirth, unadjusted and adjusted difference‐in‐difference analyses stratified by risk level. Appendix S4. Effect of the MNH+ intervention on intermediary outcomes (full study population). Appendix S5. Methods and results of sensitivity analyses. Appendix S6. Changes in place of delivery from baseline to endline by intervention status. Appendix S7. Example of the delivery room in a study facility before (A) and after (B) quality improvement activities. [file TMI-24-636-s001.docx]
